# Ratiometric pH Sensing and Imaging in Living Cells with Dual-Emission Semiconductor Polymer Dots

**DOI:** 10.3390/molecules24162923

**Published:** 2019-08-12

**Authors:** Piaopiao Chen, Iqra Ilyas, Su He, Yichen Xing, Zhigang Jin, Chaobiao Huang

**Affiliations:** 1 College of Chemistry and Life Sciences, Zhejiang Normal University, Jinhua 321004, China; 2 Xingzhi College, Zhejiang Normal University, Jinhua 321004, China

**Keywords:** Pdots, semiconductor polymer, FRET, pH sensing, cell imaging

## Abstract

Polymer dots (Pdots) represent newly developed semiconductor polymer nanoparticles and exhibit excellent characteristics as fluorescent probes. To improve the sensitivity and biocompatibility of Pdots ratiometric pH biosensors, we synthesized 3 types of water-soluble Pdots: Pdots-PF, Pdots-PP, and Pdots-PPF by different combinations of fluorescent dyes poly(9,9-dioctylfluorenyl-2,7-diyl) (PFO), poly[(9,9-dioctyl-fluorenyl-2,7-diyl)-co-(1,4-benzo-{2,1′,3}-thiadazole)] (PFBT), and fluorescein isothiocyanate (FITC). We found that Pdots-PPF exhibits optimal performance on pH sensing. PFO and FITC in Pdots-PPF produce pH-insensitive (λ = 439 nm) and pH-sensitive (λ = 517 nm) fluorescence respectively upon a single excitation at 380 nm wavelength, which enables Pdots-PPF ratiometric pH sensing ability. Förster resonance energy transfer (FRET) together with the use of PFBT amplify the FITC signal, which enables Pdots-PPF robust sensitivity to pH. The emission intensity ratio (I_517_/I_439_) of Pdots-PPF changes linearly as a function of pH within the range of pH 3.0 to 8.0. Pdots-PPF also possesses desirable reversibility and stability in pH measurement. More importantly, Pdots-PPF was successfully used for cell imaging in Hela cells, exhibiting effective cellular uptake and low cytotoxicity. Our study suggests the promising potential of Pdots-PPF as an in vivo biomarker.

## 1. Introduction

Intracellular pH is closely related to cellular behavior and pathological processes, including many types of cancer and neurodegenerative diseases [1]. Therefore, establishing and maintaining an appropriate pH in a single cell compartment is critical to its normal physiology. pH sensors have a wide range of applications such as food, beverages, environmental monitoring, chemical processing, biomedical applications, and laboratory pH measurements [2,3]. These applications typically require highly reliable and accurate pH sensors. Various techniques have been explored for pH measurement. Currently, the most common one is based on glass electrodes, which have also been recognized as commercial products with high stability and accuracy. However, they still have some drawbacks, such as the high brittleness of glass membrane electrodes, which create a strong incentive to develop new pH sensors [4,5].

Fluorescent probes have proven to be an effective tool compared to other detection techniques such as electrochemical methods because of their many advantages, including simple operation, high sensitivity, selectivity, and in situ imaging [6,7]. To date, several materials have been used for pH sensing. For examples, Zhong et al. prepared carbon dots originated from carnation for fluorescent and colorimetric pH sensing [8]. Gao et al. designed a protease-activated ratiometric fluorescent probe based on Förster resonance energy transfer (FRET) between a pH-sensitive fluorescent dye and biocompatible Fe_3_O_4_ nanocrystals [9]. Kim et al. demonstrated a novel method for the preparation of a fluorescence-based pH sensor by combining the plasmon resonance band of Ag core and pH sensitive dye (HPTS) [10]. In addition, Ag metal mid layer, graphene, and other materials were also used for pH sensing [11,12,13]. However, these materials are limited by large size and high cytotoxicity in biological application. Fluorescent semiconductor Pdots have shown significantly improved behaviors and demonstrated as a suitable candidate for pH sensing. Recently, fluorescent semiconductor Pdots have attracted great interest. Semiconductor Pdots have proven to be unique features of fluorescence sensing and imaging, including high single particle brightness, excellent photostability, and low cytotoxicity, and have been developed for sensing, imaging, and photodynamic therapy [14,15,16].

Fluorescence sensors based on single wavelengths are easily disturbed by variable environmental conditions, so fluctuations in excitation sources and inaccurate probe concentrations have severely limited their application. In order to overcome these problems, the ratiometric fluorescence method was developed [17,18]. Fluorescence proportional sensing can effectively eliminate the deviation by measuring the fluorescence intensity ratio of two or more emission bands of different wavelengths, thereby improving the detection accuracy. Compared to the single fluorescence intensity-based fluorescence sensors, ratiometric fluorescent probes are rarely affected by excitation source fluctuations, probe concentration, probe photobleaching, instrumentation, or environmental influences [19,20,21,22].

In this study, we synthesized and optimized semiconductor Pdots via selective reprecipitation of two or three dyes: poly(9,9-dioctylfluorenyl-2,7-diyl) (PFO), poly[(9,9-dioctyl-fluorenyl-2,7-diyl)-co-(1,4-benzo-{2,1′,3}-thiadazole)] (PFBT), and fluorescein isothiocyanate (FITC) with cross-linking reagent poly(styrene-co-maleic anhydride) (PSMA). Comparison among these Pdots and previously reported FITC pH sensor confirmed that Pdots-PPF exhibited optimal performance on pH sensing. Pdots-PPF emitted dual fluorescence at the wavelength of 439 nm and 517 nm when excited at 380 nm. The pH-sensitive ratiometric Pdots-PPF provides a reliable method for monitoring pH based on the ratio of the variable fluorescence intensity at 517 nm to constant fluorescence intensity at 439 nm (I_517_/I_439_). We also showed that the synthesized Pdots-PPF possesses excellent biocompatibility and cell imaging property in Hela cells.

## 2. Results and Discussion

### 2.1. Design of Ratiometric pH Sensor Pdots-PF

**Scheme 1:**
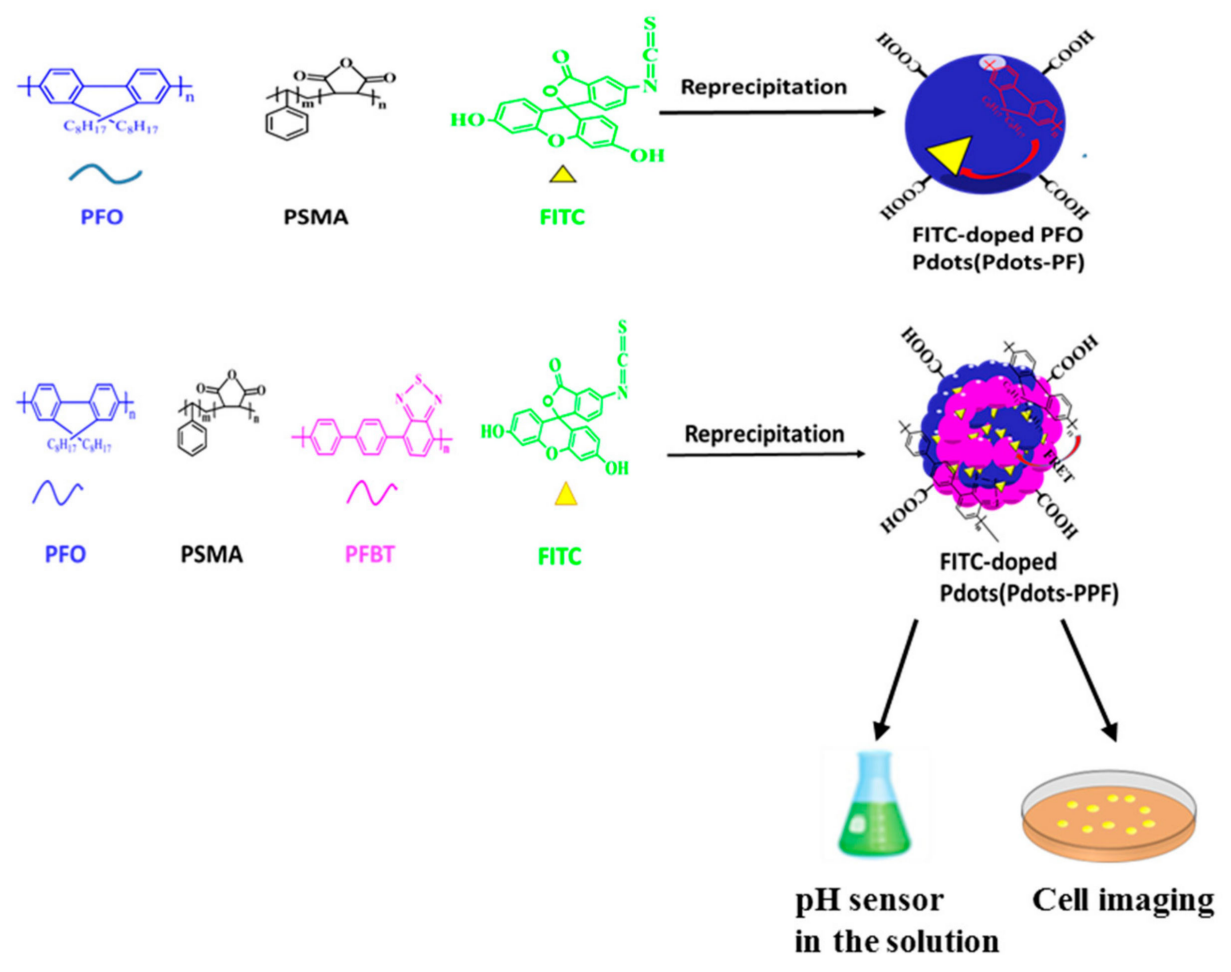
A scheme showing the preparation and application of Pdots-PF and Pdots-PPF.

**Figure 1:**
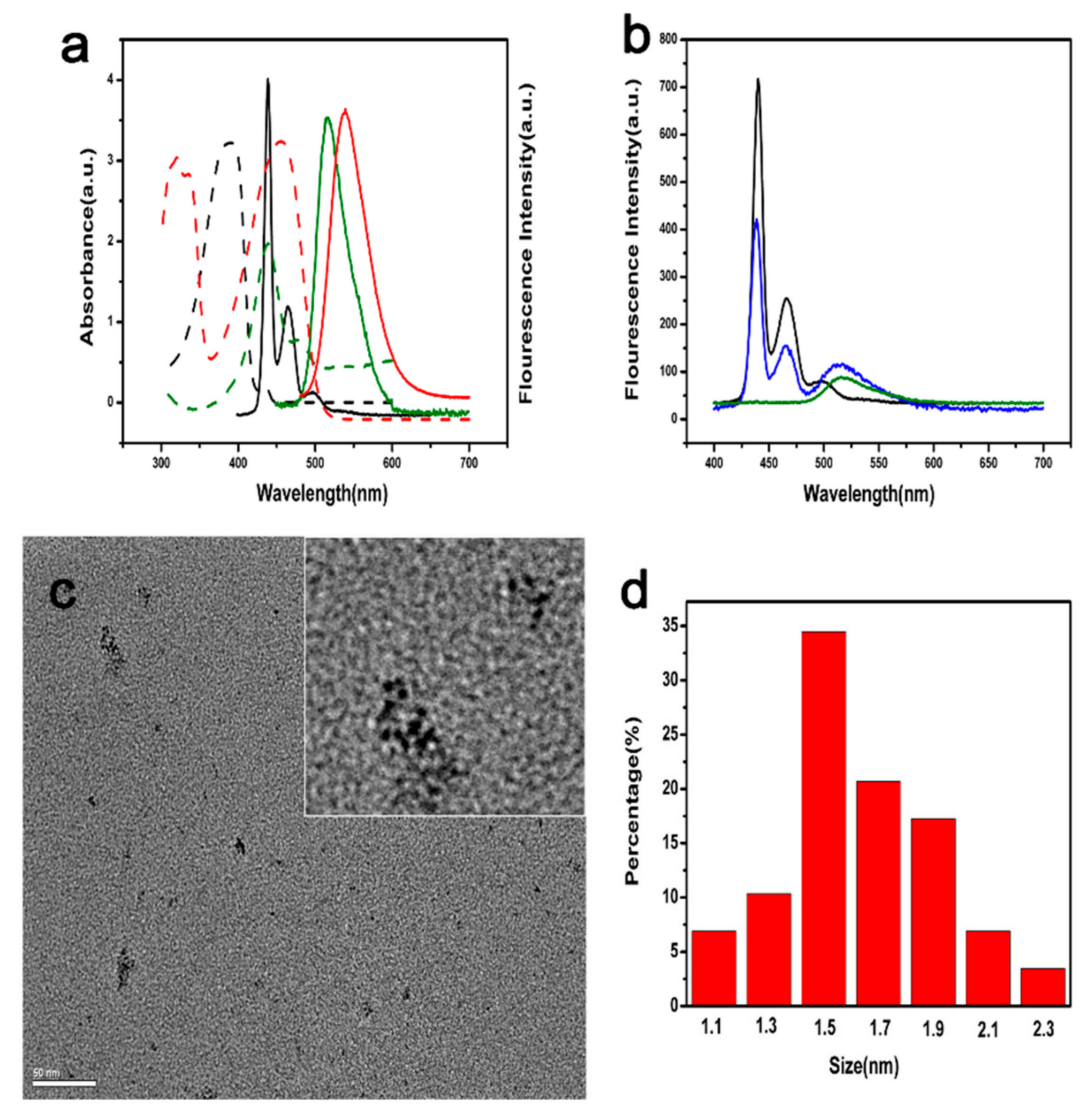
(**a**) Absorption of PFO (black dotted line), PFBT (red dotted line), and FITC (green dotted line), and emission spectra of PFO (black solid line), PFBT (red solid line), and FITC (green solid line) when excited at the optimal wavelength. (**b**) Emission spectra of PFO (black solid line), Pdots-PF (blue solid line), and FITC (green solid line) when excited at 380 nm. (**c**) TEM of Pdots-PF. Scale bar represents 50 nm. The inset shows the enlarged view of TEM of Pdots-PF. (**d**) The particle size distribution of Pdots-PF.

In order to improve currently available pH-sensitive ratiometric Pdots for better performance on pH sensing and biocompatibility, we first chose pH-insensitive dye PFO and pH-sensitive dye FITC cross-linked by PMSA to prepare dual-emission semiconductor Pdots (Pdots-PF, Scheme 1). The emission spectra of PFO (peak at 439 nm and 465 nm) and FITC (peak at 517 nm) are obviously distinct (Figure 1a), which is a prerequisite for the design of dual emission ratiometric probes. We found the significant spectral overlap between the emission spectrum of PFO and the absorption spectrum of FITC, raising the possibility of FRET from PFO to FITC (Figure 1a). As depicted in Figure 1b, the synthesized Pdots-PF showed three emission bands upon excitation at 380 nm. The intensity of Pdots-PF at 439 nm and 465 nm derived from PFO emission (blue line) was decreased as compared to that of PFO Pdots (black line), with concomitant increase of intensity at 517 nm derived from FITC emission (with same excitation at 380 nm), indicating the occurrence of FRET from PFO to FITC.

The size of semiconductor polymer nanoparticles is important for cellular uptake and cytotoxicity during biological application. We utilized transmission electron microscopy (TEM) to determine the morphology of Pdots-PF. The TEM micrographs (Figure 1c) of Pdots-PF shows well dispersed nearly spherical particles, with an average diameter of 1.5 ± 0.10 nm (Figure 1d). 

**Figure 2:**
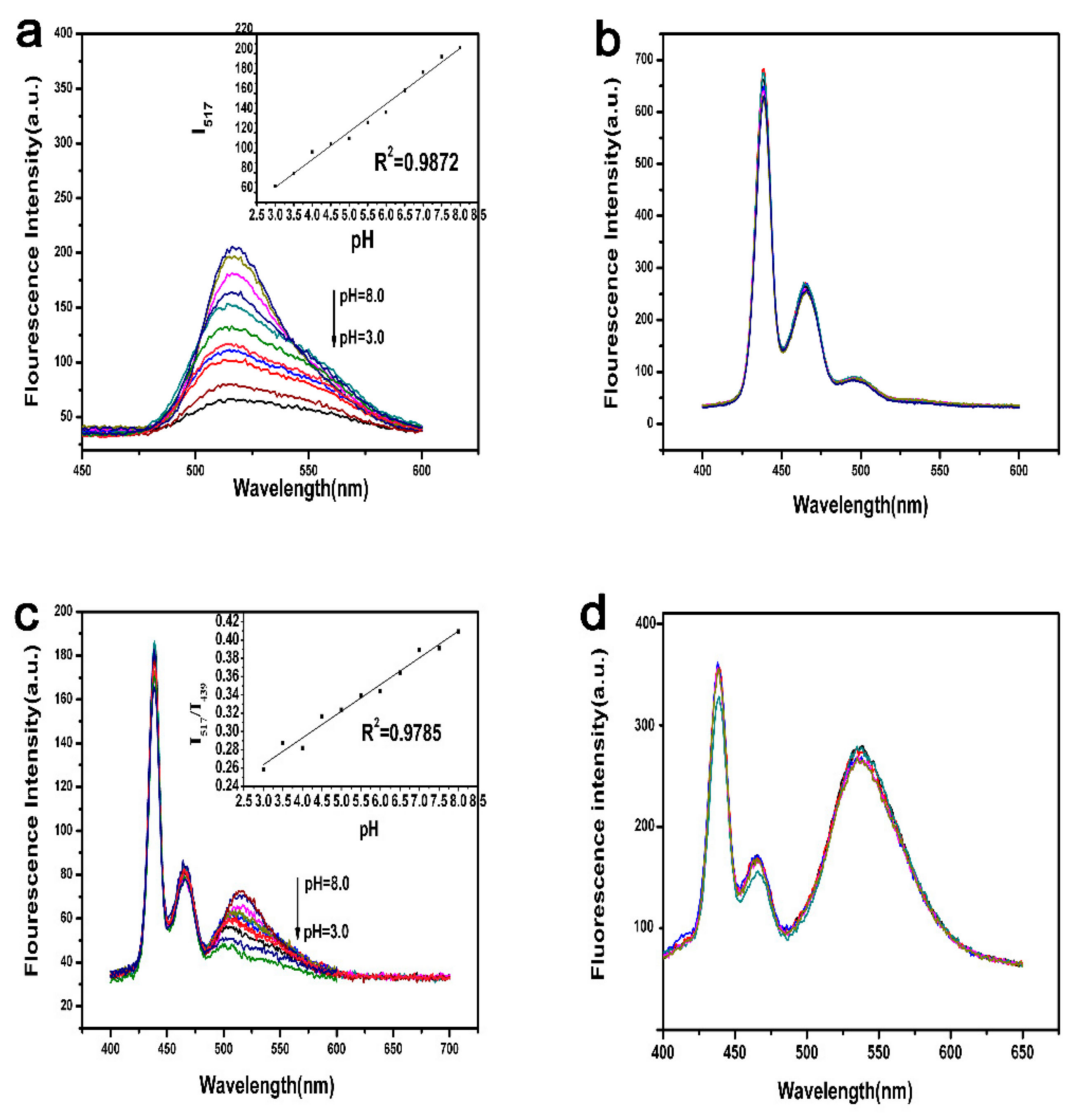
(**a**) Fluorescence emission spectra of FITC in pH 3.0–8.0 when excited at 488 nm, inset shows the linear correlation between the I_517_ and the pH. (**b**) Fluorescence emission spectra of PFO Pdots in pH 3.0–8.0 when excited at 380 nm. (**c**) Fluorescence emission spectra of Pdots-PF in pH 3.0–8.0 when excited at 380 nm, inset shows the linear correlation between the I_517_/I_439_ and the pH. (**d**) Fluorescence emission spectra of Pdots-PP in pH 3.0–8.0 when excited at 380 nm.

FITC has been applied as the pH sensor previously [23]. This was confirmed by our results (Figure 2a). However, as we mentioned earlier, the single fluorescent pH probes are easily disturbed by environment and call for ratiometric fluorescent probes. Next, we explored the dual-emission intensity changes of PFO Pdots and Pdots-PF in response to pH. The fluorescence intensity of PFO Pdots hardly changes with pH (Figure 2b). With the increasing pH from 3.0 to 8.0, the fluorescence intensity of Pdots-PF showed different models: The emission peak of FITC (517 nm) increased while that of PFO (439 nm and 465 nm) remained constant (Figure 2c). The invariable PFO emission suggested that both of the fluorescence intensity of PFO and the FRET efficiency are nonresponsive to pH. Thus, we chose the fluorescence intensity of FITC (I_517_) and PFO (I_439_) as the response signal and reference signal, respectively. As shown in inset of Figure 2c, we characterized the emission ratio I_517_/I_439_ of Pdots-PF. However, the linear relationship of I_517_/I_439_ to pH is not ideal in the range of pH 3.0 to 8.0, and the correlation coefficient is 0.9785. We speculate that this is probably caused by the relatively weak signal of FITC upon excitation at 380 nm, thus the pH-dependent change of FITC intensity is subtle. 

### 2.2. Design of Ratiometric pH Sensor Pdots-PPF

In order to address the above issue and improve the performance of Pdots-PF on pH sensing, we try to introduce PFBT, a polymer dye with similar emission wavelength of FITC (Figure 1a), to amplify the FITC signal and prepare Pdots-PPF, which contains fluorescent dyes PFO, PFBT, FITC, and cross-linking reagent PSMA.

**Figure 3:**
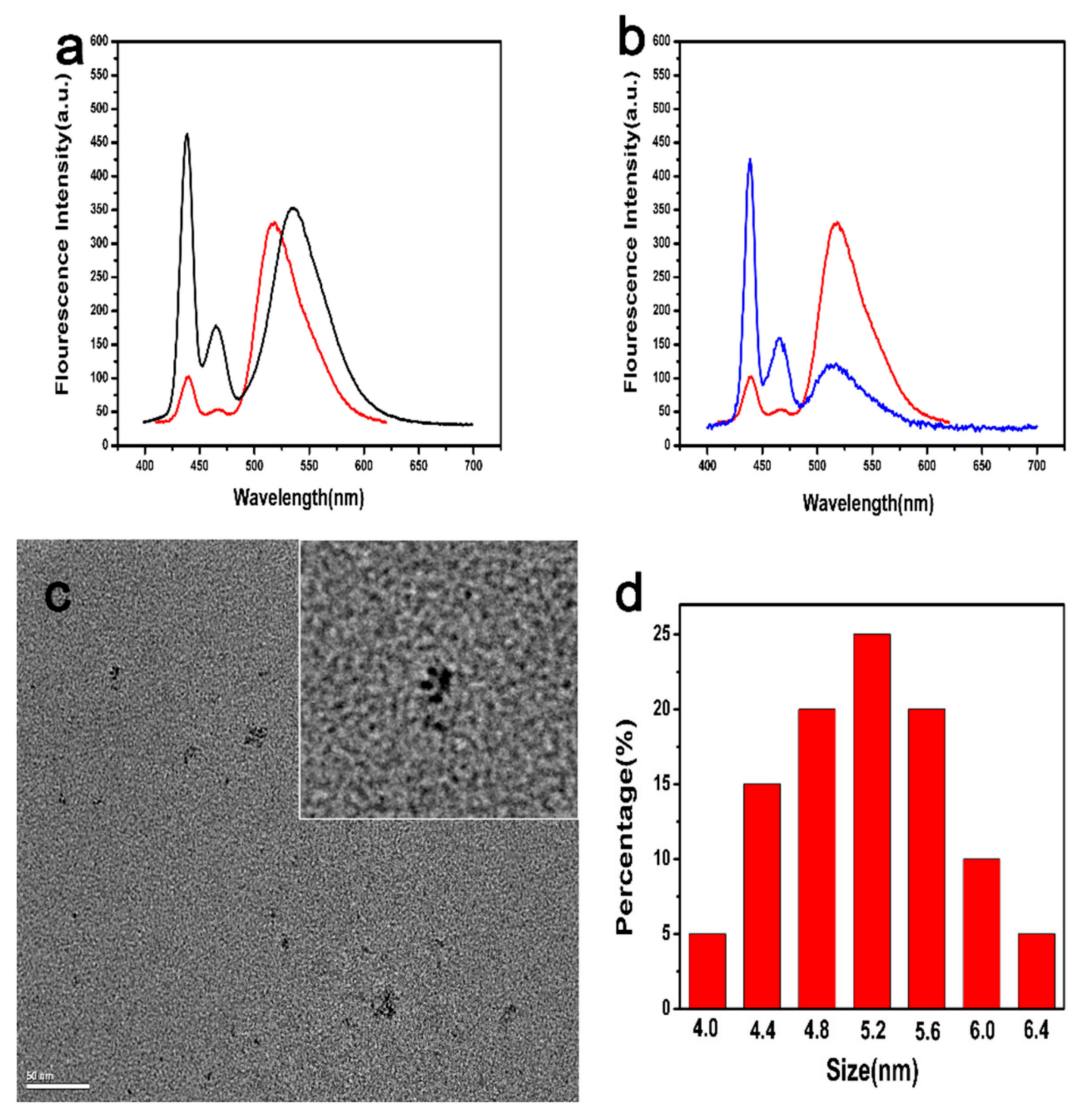
(**a**) Fluorescence spectra of Pdots-PP (black solid line) and Pdots-PPF (red solid line) when excited at 380 nm. (**b**) Fluorescence spectra of Pdots-PF (blue solid line) and Pdots-PPF (red solid line) when excited at 380 nm. (**c**) TEM of Pdots-PPF. Scale bar represents 50 nm. The inset shows the enlarged view of TEM of Pdots-PPF. (**d**) The particle size distribution of Pdots-PPF.

Pdots-PPF was synthesized according to Scheme 1. As comparison, we also prepared Pdots-PP, which contains PFO, PFBT, and PSMA. The spectral overlap between the emission spectrum of PFO and the absorption spectrum of PFBT indicates the potential FRET from PFO to PFBT (Figure 1a). We examined the pH response of Pdots-PP and confirmed that Pdots-PP is pH nonresponsive in the range of pH 3.0 to 8.0 (Figure 2d). We compared the fluorescence spectra of Pdots-PP and Pdots-PPF (Figure 3a), and found that the addition of FITC to Pdots-PP caused: (1) A blue-shift from 539 nm to 517 nm. Therefore, the emission peaks of PFBT and FITC were merged at 517 nm and FITC signal was successfully amplified in Pdots-PPF. (2) A decreased fluorescence intensity of PFO at 439 nm and 465 nm, indicating that similar to Pdots-PF, Pdots-PPF also showed efficient FRET from PFO to FITC/PFBT. 

Comparison of fluorescence spectra of Pdots-PF and Pdots-PPF confirmed that the weak FITC signal in Pdots-PF is replaced by a merged and amplified FITC/PFBT signal in Pdots-PPF (Figure 3b). The TEM images show that Pdots-PPF exhibits highly spherical monodispersity with a diameter of approximately 5.2 ± 0.20 nm (Figure 3c,d). We summarized the composition of Pdots-PPF and the roles of each component in Table 1, and the comparison of pH sensors used in this study in Table 2.

### 2.3. Ratiometric Fluorescence Detection of pH in Buffer Solution

**Figure 4:**
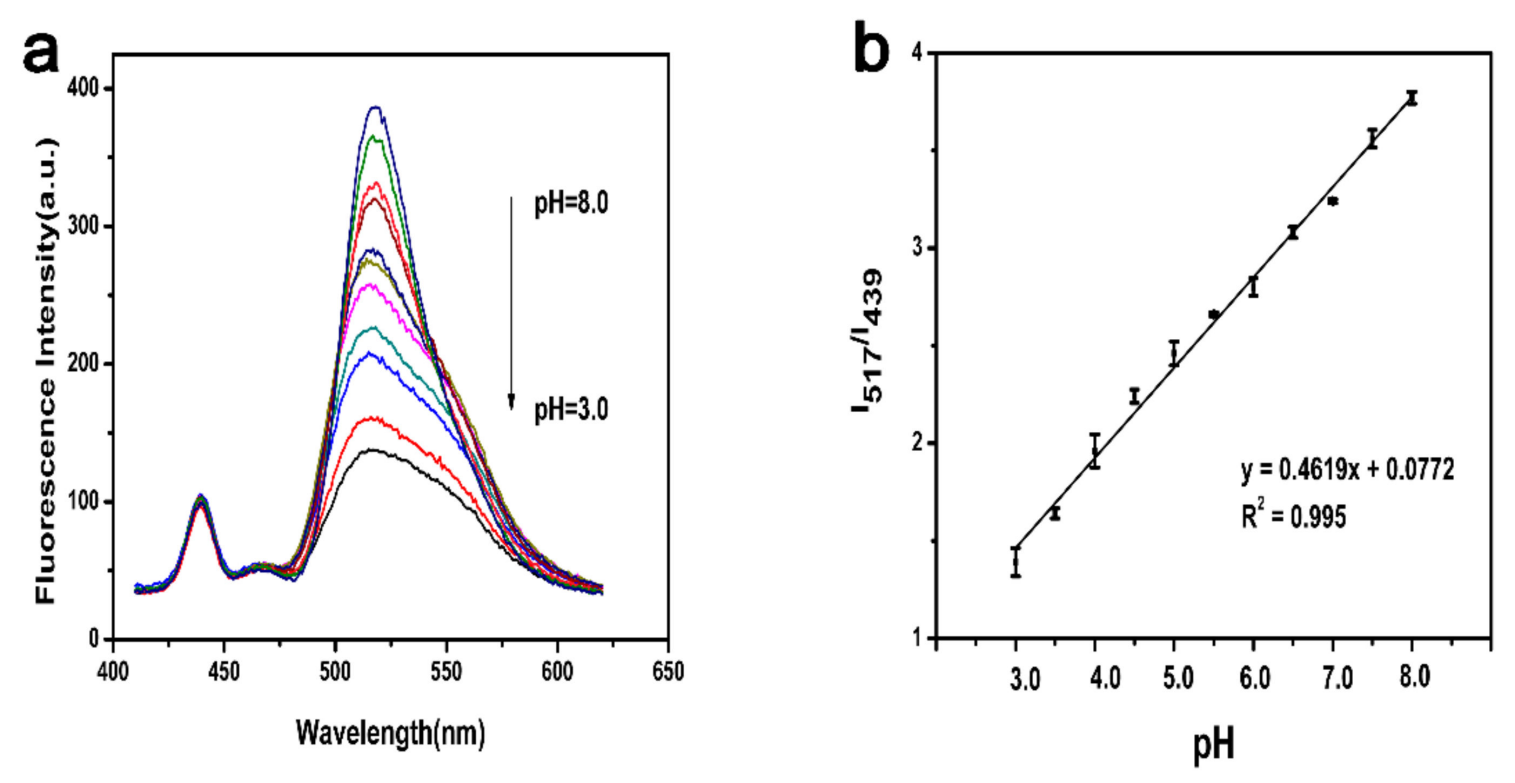
(**a**) Fluorescence emission spectra of Pdots-PPF at pH 3.0–8.0 in 20.0 mM HEPES buffer solution when excited at 380 nm. (**b**) Plot of the relative fluorescence intensity ratios (I_517_/I_439_) of Pdots-PPF in response to pH values.

We speculated that the merged FITC/PFBT signal remains pH-sensitive and PFO signal remains pH-insensitive in Pdots-PPF. To test this possibility, the pH sensitivity and ratiometric property of Pdots-PPF were examined in HEPES buffer solutions with various pH values. As we expected, the fluorescence intensity of Pdots-PPF at 517 nm increased when the pH was increased from 3.0 to 8.0 and fluorescence intensity at 439 nm is relatively constant (Figure 4a). Therefore, the pH-dependent change of FITC intensity is significantly improved in Pdots-PPF (pH 8.0 vs. pH 3.0, fold change of I_517_ is 2.77) as compared to Pdots-PF (pH 8.0 vs. pH 3.0, fold change of I_517_ is 1.66). 

Next, we examined the relationship between the ratio of the emission intensities of FITC (I_517_) to PFO (I_439_) and the pH values (Figure 4b). The change in fluorescence ratio of I_517_/I_439_ shows a good linear relationship with the pH values. The linear equation is y = 0.6149x+0.0772, and the correlation coefficient is 0.995. However, the linear relationship is lost when the pH values are beyond the range of pH 3.0 to 8.0 (data not shown). We conclude that Pdots-PPF exhibits significant advantage over Pdots-PF and FITC as a ratiometric pH sensor and can be applied for pH detection within the linear range between pH 3.0 and 8.0 (Table 2).

### 2.4. Reversibility, Stability, and Selectivity of the pH Sensors

**Figure 5:**
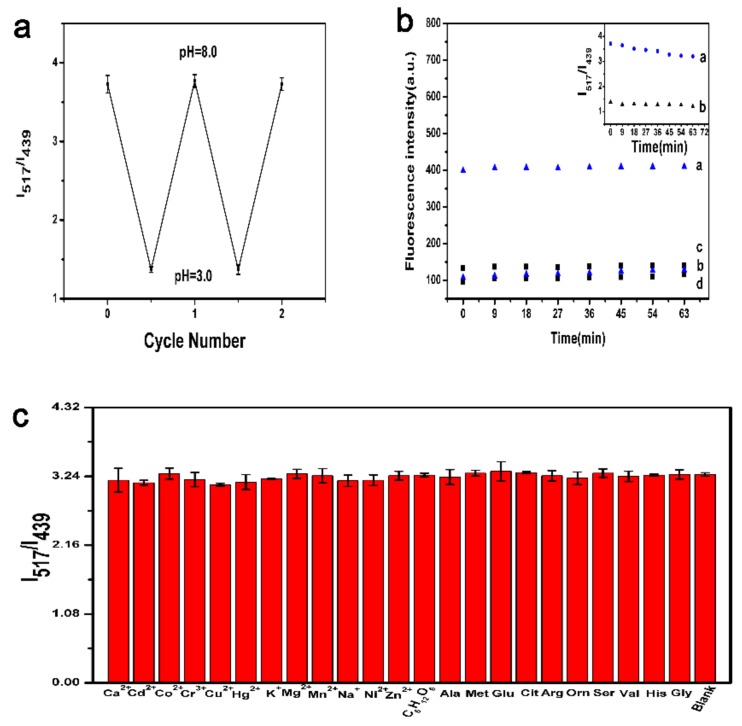
(**a**) Fluorescence intensity ratios (I_517_/I_439_) of Pdots-PPF upon repeatedly switching pH from 3.0 to 8.0. (**b**) Fluorescence intensity of λ_517_ (a,c) and λ_439_ (b,d) emission of Pdots-PPF in 20.0 mM HEPES buffer solution at pH 8.0 and 3.0. The inset: The corresponding time-dependent intensity ratios (I_517_/I_439_) of Pdots-PPF at pH = 8.0 (a) and 3.0 (b). (**c**) Selectivity of the Pdots-PPF.

The reversibility of Pdots-PPF was examined by cyclic detection of pH at pH 3.0 and 8.0 (Figure 5a). Pdots-PPF shows excellent reversibility between pH 3.0 and 8.0, which is attributed to the inherent characteristic of the fast protonation/deprotonation process of FITC and the desirable stability of the fluorescent probe. 

Furthermore, the photostability of Pdots-PPF at different pH values were investigated. Figure 5b shows the individual time-dependent fluorescence intensity of the emission at 517 nm (a,c) and 439 nm (b,d) of Pdots-PPF at different pH values under 365 nm UV lamp illumination. In summary, both the individual fluorescence intensity and the ratio of Pdots-PPF in different pH buffers (Figure 5b inset) did not show significant changes with duration of 63 min, indicating that Pdots-PPF exhibited outstanding photostability and could be used for long term monitoring of pH.

Selectivity is an important aspect in the application of pH sensors. In order to investigate the anti-interfering ability of Pdots-PPF for pH sensing, the variations in fluorescence intensity ratios (I_517_/I_439_) in the presence of potential interfering substances including ions and amino acids were examined under the same conditions. As depicted in Figure 5c, compared to the blank I_517_/I_439_ ratio in the absence of interfering substances, no detectable variations in the I_517_/I_439_ ratios were observed while adding different interfering species such as 100 μM of Ca^2+^, Cd^2+^, Co^2+^, Cr^3+^, Cu^2+^, Hg^2+^,K^+^, Mg^2+^, Mn^2+^, Na^+^, Ni^2+^, Zn^2+^, and 10 μM of glucose, alanine(Ala), methionine (Met), glutamic acid (Glu), citrulline(Cit), L-arginine (Arg), ornithine (Orn), serine (Ser), valine (Val), histidine (His), glycine (Gly) in HEPES (20 mM pH 7.0). The anti-interfering experiment reveals that Pdots-PPF displays the capacity for pH determination with high selectivity.

### 2.5. Cell Imaging Applications of Pdots-PPF

**Figure 6:**
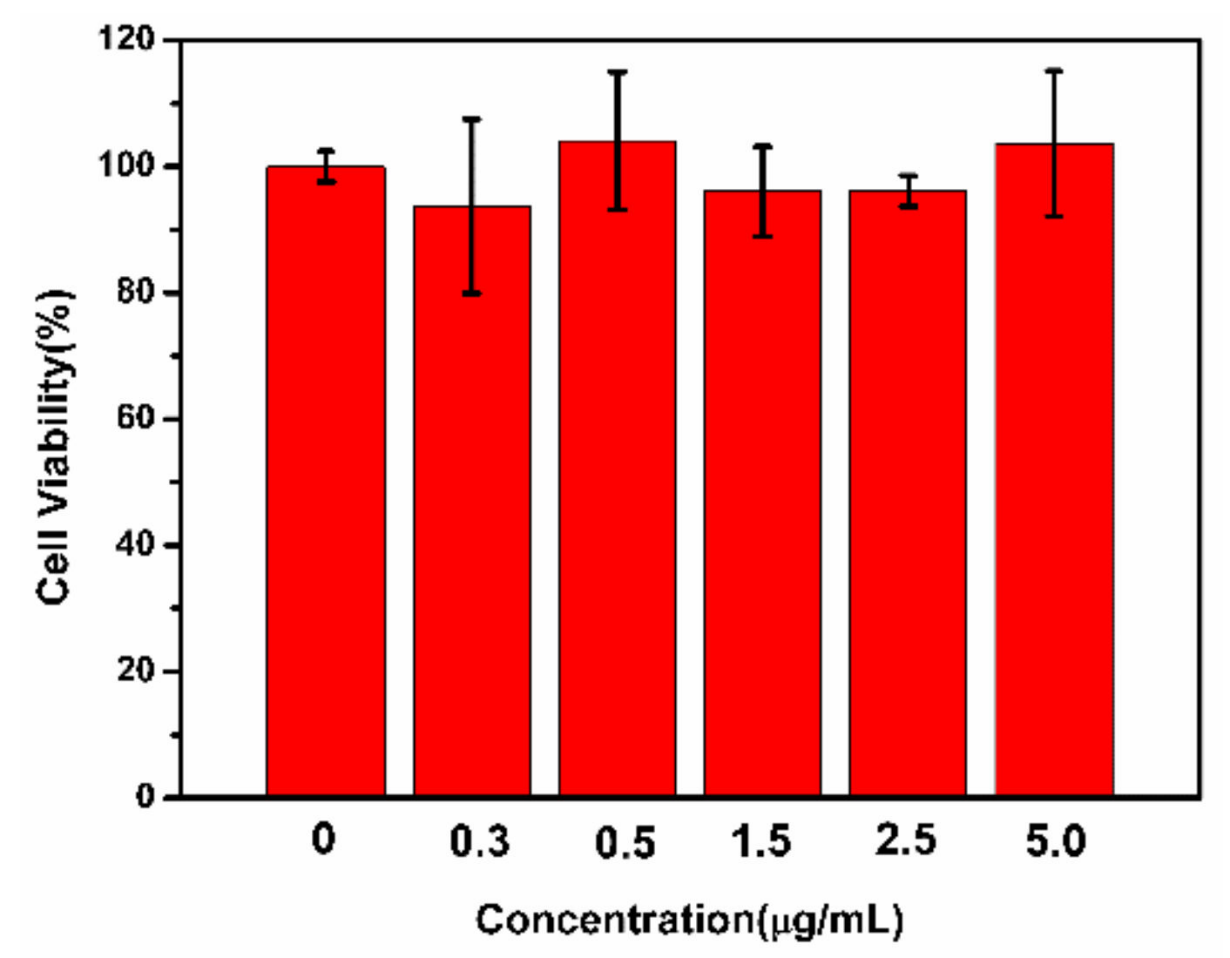
MTT based cytotoxicity assay of Hela cells after 24 h incubation with different concentrations of Pdots-PPF (0, 0.3, 0.5, 1.5, 2.5, and 5.0 μg·mL^−1^) (mean ± SD, n = 3).

The cell viabilities were examined upon exposure to the Pdots-PPF of different concentrations. Figure 6 illustrates that the Pdots-PPF exhibited extremely low cytotoxicity, with cell viability over 93% at the concentration of 0.3, 0.5, 1.5, 2.5, 5.0 μg·mL^−1^ Pdots-PPF after incubation for 24 h. 

**Figure 7:**
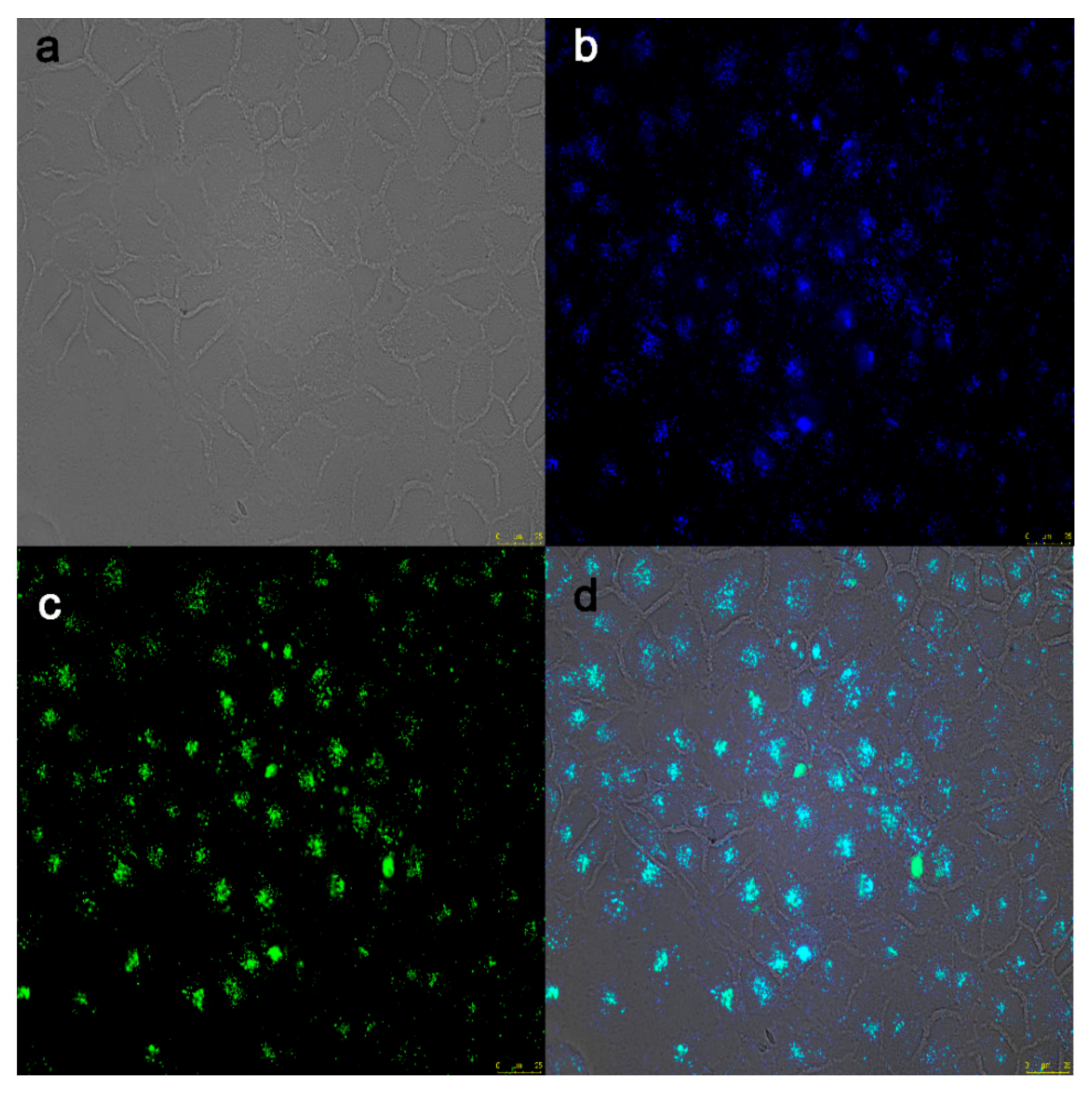
(**a**) Bright-field and (**a**–**c**) fluorescent images of Hela cells incubated with Pdots-PPF. (**d**) merged image of (**a**), (**b**) and (**c**). Scale bar represents 25 μm.

In order to investigate the potential of Pdots-PPF for intracellular pH measurements, we incubated Pdots-PPF with live Hela cells to allow cellular uptake via endocytosis. After cellular uptake, we washed Hela cells to remove extracellular Pdots-PPF. The bright-field and fluorescent images of Hela cells with intracellular Pdots-PPF are depicted in Figure 7. The Hela cells labeled with Pdots-PPF exhibited bright blue and green fluorescence under ultraviolet (340–380 nm) and blue (450–490 nm) light excitation, respectively. Close observations revealed that the Pdots-PPF was distributed mainly within the nucleus, while cell membrane and the cytoplasmic area had only weak fluorescence. 

The above experiments indicate that the Pdots-PPF is featured with photostabilities, high resistance to photobleaching, inertness to interference of metal ions and biomolecular species, and excellent biocompatibility, suggesting Pdots-PPF to be a promising candidate for bioimaging and pH biosensor applications under physiological or pathological conditions.

## 3. Experimental Section

### 3.1. Materials and Reagents

Alanine (Ala), methionine (Met), glutamic acid (Glu), citrulline (Cit), L -arginine (Arg), ornithine(Orn), serine(Ser), valine(Val), histidine(His), glycine (Gly), 2-(4-(2-hydroxyethyl)-1-piper-azinyl) ethanesulfonic acid buffer (HEPES,) and fluorescein isothiocyanate (FITC) were purchased from Aladdin (Shanghai, China). Poly(9,9-dioctylfluorenyl-2,7-diyl) (PFO), Poly[(9,9-dioctyl-fluorenyl-2,7-diyl)-co-(1,4-benzo-{2,1′,3}-thiadazole)] (PFBT) and poly(styrene-co-maleic anhydride) (PSMA) were purchased from Sigma-Aldrich (MO, USA). Tetrahydrofuran (THF), glucose, NaCl, KCl, CoCl_2_·6H_2_O, CaCl_2_·2H_2_O, MnCl_2_·4H_2_O, CdCl_2_·2.5H_2_O, ZnCl_2_, NiCl_2_·6H_2_O, CuCl_2_·2H_2_O, MgCl_2_·6H_2_O, CrCl_3_·6H_2_O, and Hg(NO_3_)_2_ were purchased from Dongnan Chemical Reagent (Zhejiang, China). All reagents were used as received without further purification.

### 3.2. Instruments

The morphology of Pdots was characterized by transmission electron microscopy (TEM) using a JEM-2100F TEM facility. The UV-vis absorption spectrum was obtained with a LZMBDA 950 spectrophotometer. Fluorescence (FL) spectra were recorded using a RF-5301PC fluorescence spectrophotometer (Shimadzu, Japan). Fluorescence were observed and imaged on a Leica DM4B fluorescence microscope. Absorbance of samples for cytotoxicity assay was measured by microplate reader (Bio-Rad iMark Microplate Absorbance Reader, Hercules, CA, USA).

### 3.3. Preparation of Pdots-PF, Pdots-PP, and Pdots-PPF

Pdots were synthesized according to a reported method [22]. FITC and polymers PFO, PFBT, and PSMA were dissolved in anhydrous THF to obtain 1.0 mg·mL^−1^ stock solutions under sonication, respectively.

To prepare Pdots-PF, the solutions of PFO, PSMA, and FITC in THF were mixed to make a solution mixture with PFO concentration of 30 μg·mL^−1^, PSMA concentration of 16 μg·mL^−1^, and FITC concentration of 60 μg·mL^−1^. A 3 mL quantity of the solution mixture was quickly injected into 10 mL of ultrapure water and sonicated for 5 min, and then THF was removed by reduced pressure distillation to get a colloidal solution of Pdots-PF. After that, the as-prepared colloidal solution was filtered through a 0.22 μm membrane filter to remove larger aggregates. The prepared Pdots-PF solution was stored at 4 °C.

To prepare Pdots-PP, the solutions of PFO, PFBT, and PSMA were mixed to make a solution mixture with PFO concentration of 30 μg·mL^−1^, PFBT concentration of 20 μg·mL^−1^, and PSMA concentration of 16 μg·mL^−1^. The subsequent processing is the same as Pdots-PF. The Pdots-PP concentration was 15 μg·mL^−1^ and then the prepared Pdots-PP solution was stored at 4 °C.

To prepare Pdots-PPF, the solutions of PFO, PFBT, PSMA, and FITC in THF were mixed to make a solution mixture with PFO concentration of 30 μg·mL^−1^, PFBT concentration of 20 μg·mL^−1^, PSMA concentration of 16 μg·mL^−1^, and FITC concentration of 60 μg·mL^−1^. The subsequent processing is the same as Pdots-PF. The Pdots-PPF concentration was 15 μg·mL^−1^ and then the prepared Pdots-PPF solution was stored at 4 °C.

### 3.4. Procedures for pH Detection

An equivalent amount of 20 μL of Pdots (15 μg·mL^−1^) solution was put into a series of 2 mL centrifuge tubes and diluted to 1 mL using HEPES buffers (20 mM) with various pH values, respectively. The fluorescence measurements were performed with 380 nm light excitation for Pdots and 488 nm light excitation for FITC.

### 3.5. Cell Culture and Cell Imaging of Pdots

Hela cells (CCL-2, epithelial of human cervical cancer cells, ATCC, Manassas, VA, United States) were maintained in DMEM supplemented with 10% fetal bovine serum, penicillin, and streptomycin, at 37 °C in a humidified, 5% CO_2_ atmosphere. Hela cells were treated with 3.0 μg·mL^−1^ Pdots. After incubation for 24 h in the dark, Hela cells were washed with PBS buffer for 3 times and harvested for cell imaging with Leica DM4B fluorescence microscope and the LAS X software (Leica Microsystems, Wetzlar, Germany). 

### 3.6. Cytotoxicity Assay

Hela cells were treated with indicated concentrations of Pdots for 24 h. Ten (10) μL of 3-(4,5-dimethythiazol-2-yl)-2,5-diphenyl tetrazolium bromide (MTT) was added. Cells were incubated at 37 °C for 4 h and then lysed by 100 μL of lysis buffer (4% Triton X-100 and 0.14% HCl in 2-propanol). Absorbance at 595 nm was measured on a microplate reader (Bio-Rad, Hercules, CA, USA).

## 4. Conclusions

In general, we optimized semiconductor Pdots and obtained Pdots-PPF with optimal behavior for pH detection. Each component of Pdots-PPF is indispensable and plays an important role in ratiometric pH sensing. Ultimately, Pdots-PPF can be successfully applied for cell imaging, indicating its promising potential for in vivo bioimaging and biosensing, which is currently under our investigation. 

## Figures and Tables

**Table 1:** The composition of Pdots-PPF and the roles of each component.

Components	Functions
PFO	FRET donor, provide reference signal (I_439_)
PFBT	FRET acceptor, FITC signal amplifier
FITC	FRET acceptor, provide pH-responsive signal (I_517_)
PMSA	Cross-linking

**Table 2:** Comparison of pH sensors used in this study.

pH Sensors	Components	FRET	Emission	pH Sensitivity
FITC	FITC	No	Single	Robust
PFO Pdots	PFO, PMSA	No	Single	No
Pdots-PF	PFO, FITC, PMSA	PFO to FITC	Dual	Weak
Pdots-PP	PFO, PFBT, PMSA	PFO to PFBT	Dual	No
Pdots-PPF	PFO, PFBT, FITC, PMSA	PFO to FITC/PFBT	Dual	Robust
